# Intriguingly high thermal conductivity increment for CuO nanowires contained nanofluids with low viscosity

**DOI:** 10.1038/s41598-018-23174-z

**Published:** 2018-03-27

**Authors:** Dahai Zhu, Lingling Wang, Wei Yu, Huaqing Xie

**Affiliations:** 1 School of Environmental and Materials Engineering, College of Engineering, Shanghai Polytechnic University, Shanghai, 201209 China; 2 Shanghai Innovation Institute for Materials, Shanghai, 200444 China

## Abstract

Nanofluids offer the exciting new possibilities to enhance heat transfer performance. In this paper, experimental and theoretical investigations have been conducted to determine the effect of CuO nanowires on the thermal conductivity and viscosity of dimethicone based nanofluids. The CuO nanowires were prepared through a thermal oxidation method, and the analysis indicated that the as-prepared CuO nanowires had high purity, monocrystalline with a monoclinic structure and large aspect ratio compared to CuO nanospheres. The experimental data show that the thermal conductivity of the nanofluids increases with the volume fraction of CuO nanowires or nanospheres, with a nearly linear relationship. For the nanofluid with the addition of 0.75 vol.% CuO nanowires, the thermal conductivity enhancement is up to 60.78%, which is much higher than that with spherical CuO nanoparticles. The nanofluids exhibit typical Newtonian behavior, and the measured viscosity of CuO nanowires contained nanofluids were found only 6.41% increment at the volume fraction of 0.75%. It is attractive in enhanced heat transfer for application. The thermal conductivity and viscosity of CuO nanofluids were further calculated and discussed by comparing our experimental results with the classic theoretical models. The mechanisms of thermal conductivity and viscosity about nanofluids were also discussed in detail.

## Introduction

The concept of “nanofluid” was proposed by Choi^1^. Nanofluids are the suspensions of solid nanoparticles, which are made of mixing the nanoparticles in various base liquids such as water, thermal oils, dimethicone or ethylene glycol. For the past decades, nanofluids have received much attention due to their enhanced heat transfer^2^. Nanofluids offer the exciting new possibilities to enhance heat transfer performance compared to the pure liquids, so it can be considered to be the next generation heat transfer fluids^3^. Compared with the conventional solid–liquid suspensions for enhancing heat transfer, nanofluids not only have unique thermal transport properties, but also they have some superior performances that are unavailable in traditional heat transfer fluids^1,4^. The relative higher surface area of nanoparticles significantly improves heat transfer capabilities, and it increases the stability of the suspensions. In addition, the abrasion-related properties can be improved by nanofluids^5^.

Metal oxides are commonly used as thermal additives in nanofluids, due to their outstanding properties such as high thermal conductivity, electrical insulation, excellent compatibility with base fluid and high cost performance ratio^6^. Al_2_O_3_, TiO_2_, ZnO and CuO are the most popular metal oxide nanoparticles. Nanofluids containing metal oxides have exhibited special potentials in heat transfer applications. These advantages may be applied in some areas. María *et al*. reported an experimental work on thermal conductivity and viscosity measurements of ethylene glycol-based Al_2_O_3_ nanofluids^7^, and the results showed a considerable 19% enhancement on thermal conductivity. Vasheghani *et al*. used the hot wire method to measure the thermal conductivity of micro and nanofluids.When 3 wt.% of TiO_2_was added, they found that a maximum enhancement of 57% using TiO_2_/engine oil nanofluids as the heat transfer media^8^. Yu *et al*. have made an investigation on the thermal conductivity and viscosity of ethylene glycol based ZnO nanofluid^9^, they found that the thermal conductivity of ZnO-EG nanofluids depended strongly on particle concentration, and it increases nonlinearly with the volume fraction of the nanoparticles. The enhanced value of 5.0 vol.% ZnO-EG nanofluid is 26.5%. Among various metal oxide thermal additives, copper oxide and copper-based oxide materials have aroused widespread concern. Copper oxide (CuO) is one of the research hot spots and CuO has its unique advantages^10,11^. Compared to Al_2_O_3_, TiO_2_ and ZnO, CuO has higher thermal conductivity. CuO is a monoclinic crystal structure and it has many attractive properties, a p-type semiconductor with a narrow band gap (1.2 eV)^12^. The nano-CuO material has great potential applications in heterogeneous catalyst, battery anode material, photothermal, photoconductive materials and other fields because of its attractive characteristics such as light^13^, electricity, magnetism and catalysis^14–16^. When it is used as a nanofluid additive, it will show excellent performance, and can be used for heat transfer applications due to its enhancement in thermal conductivity. Sivakuma *et al*. had made a series of experimental investigations in thermal conductivity of low volume percentage of CuO ethylene glycol nanofluid^17^, and they declared that there was a considerable enhancement in the thermal conductivity.

Experimental studies and theoretical predictions prove that one-dimensional materials are more likely to form thermal pathways^18^. Up to now, copper oxide nanoparticles have been used as the nanofluids heat transfer additive in most of the literatures, but few study concerns about copper oxide nanowires. In this paper, we want to prove and verify the effect of copper oxide nanowires on the thermal conductivity of nanofluids. We prepared CuO nanowires and spherical nanoparticles, and then CuO/dimethicone nanofluids were prepared by a two-step method. We found intriguingly high thermal conductivity increment of nanofluids at low loading using CuO nanowires as the thermal additive. The transport properties including thermal conductivity and viscosity were measured. The effects of the particle volume fraction, shape of the additive, mechanisms and theoretical model on the thermal conductivity were further investigated.

## Results and Discussions

### Preparation and growth mechanism of copper nanowires

To date, various morphology and structure of CuO particles were synthesized, such as copper-based materials nanowires, nanospherical, nanoflower, and they have been extensively investigated worldwide. CuO nanostructure materials were usually used as additive fillers to improve the thermal properties of nanofluids with different base fluid. Water is a perfect fluid for heat transfer applications because of its favorable thermophysical properties, but the boiling point of water is low, which means that it cannot be applied to higher temperatures. Dimethicone is usually used as heat conducting oil. However,it has very low thermal conductivity, so a lot of efforts have been made to increase its thermal conductivity. In this work, it was selected as the base liquid due to its higher boiling point compared to other base liquids such as water, EG or their mixtures. Moreover, dimethicone is non-toxic, with physiological inertia, good chemical stability, electrical insulation, low freezing point and hydrophobic performance. It can be used in the range of 50~180 °C. Currently, few studies have been reported to investigate the thermal conductivity of dimethicone based nanofluids containing CuO nanoparticles.

**Figure 1:**
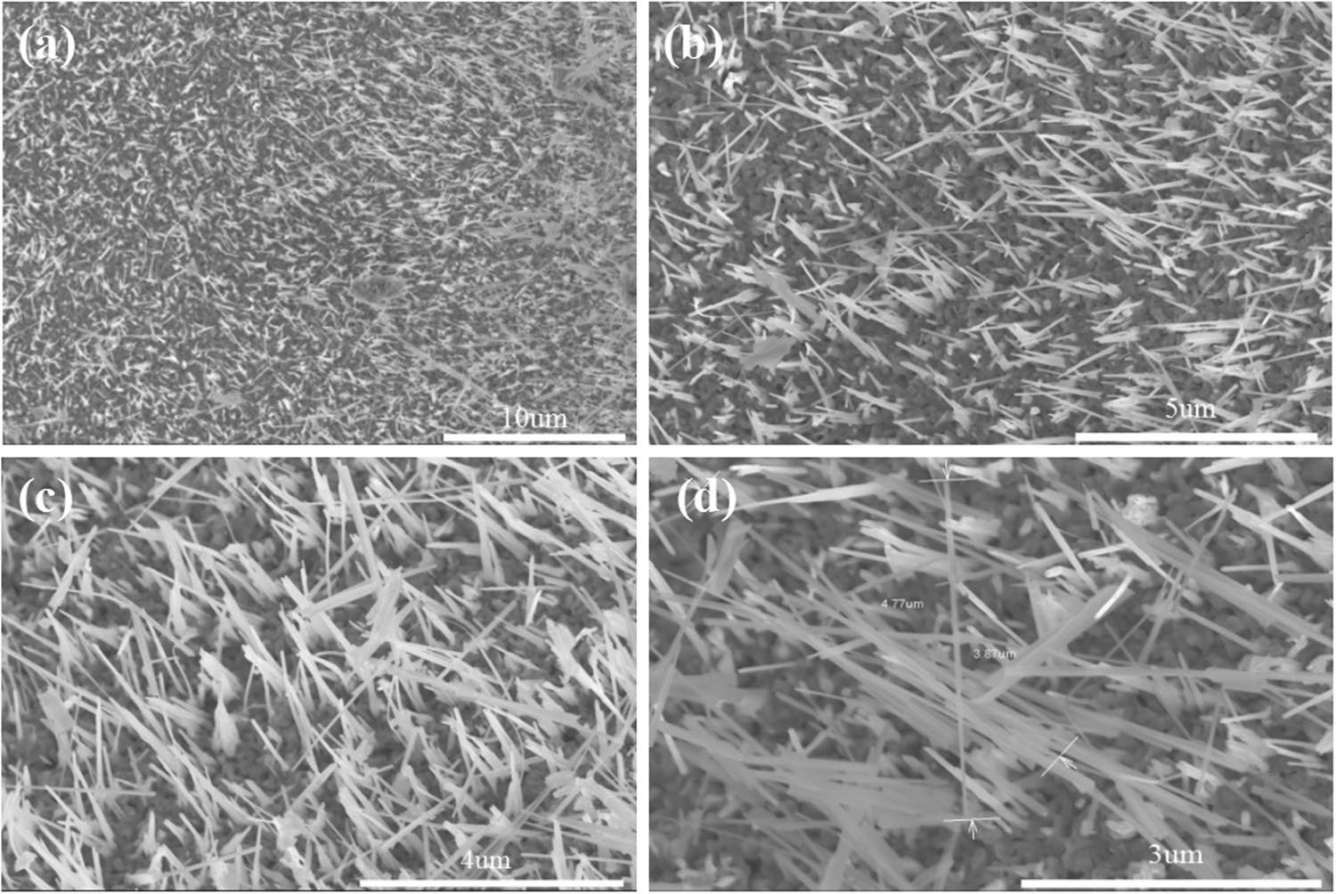
Typical SEM images of CuO nanowires at different magnification.

**Figure 2:**
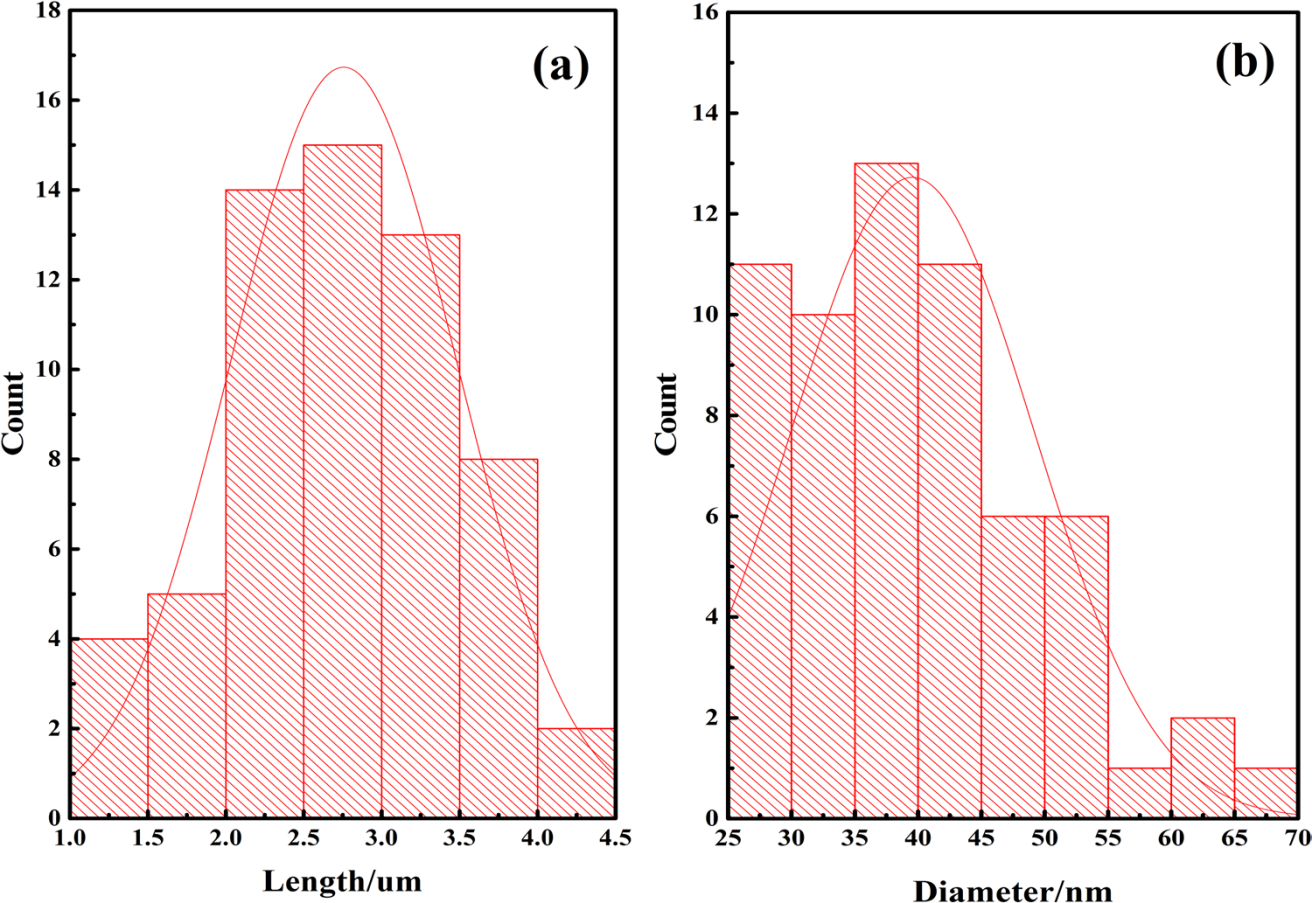
Statistic of the length and diameter of the copper oxide nanowires.

The CuO nanowires were successfully synthesized on the Cu substrate by heating Cu foils in air. Figure 1 shows the typical scanning electron microscopy images of CuO nanowires. A large amount of CuO nanowires can be observed clearly. The as-synthesized CuO nanowires display wire-like structure with diameters varying from 30 to 80 nm and length from 3.5 to 5.5 um. Combined with Fig. 1(c,d), we made a statistic of the length and diameter of copper oxide nanowires and drew the positive distribution curve. It can be seen from Fig. 2 that the optimal length and diameter are 2.79 um and 39.12 nm, respectively.

**Figure 3:**
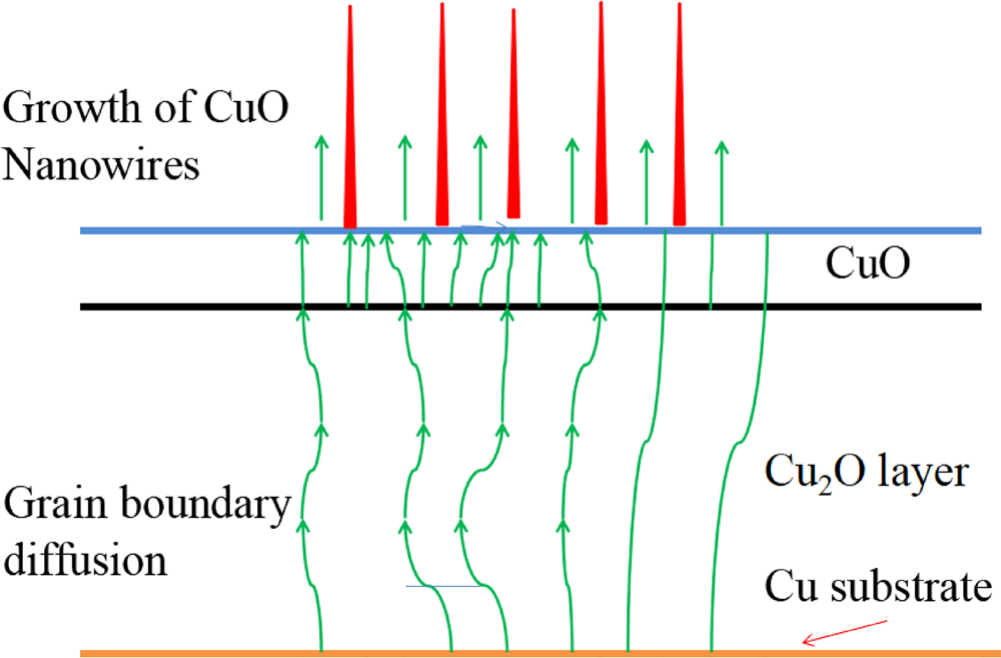
Growth mechanism of CuO nanowires^62^.

As reported in our previous paper^19^, the as-synthesized CuO structures are microspheres with the diameter about 1 μm. The surface of the spheres is rough with some gullies. Figure 3 shows the growth mechanism of CuO nanowires. From top to bottom are: CuO nanowires, CuO layer, Cu_2_O layer,and Cu substrate. The Cu_2_O layer is much thicker than CuO layer. During the growth of CuO layer and CuO nanowires, Cu ions diffuse upward through the Cu_2_O layer^20^. Temperature has an important effect on the growth of CuO nanowires, the main diffusion way of Cu ion is grain boundary diffusion at the temperature of 400 °C^21^ in this paper. In the process of grain boundary diffusion (Fig. 3), copper ions diffuse to the surface and continue to diffuse along the grain boundary of the surface grain or nucleation at the surface grain boundary. Copper ions spread along the grain boundaries of the nuclei after nucleation, resulting in CuO nanowires along the grain boundary, and then a CuO layer can form on the Cu_2_O layer^22,23^.

As a matter of fact, more and more CuO molecules are generated and forming a monoclinic CuO critical core with the continuation of the oxidation reaction^24–26^. The crystal theory shows that the core shape of the monoclinic crystal is usually a pointed rod-like structure, so most of the copper ions are transported to the tip, only a small part of the radial growth, therefore, it will form a one-dimensional linear structure^27,28^.

**Figure 4:**
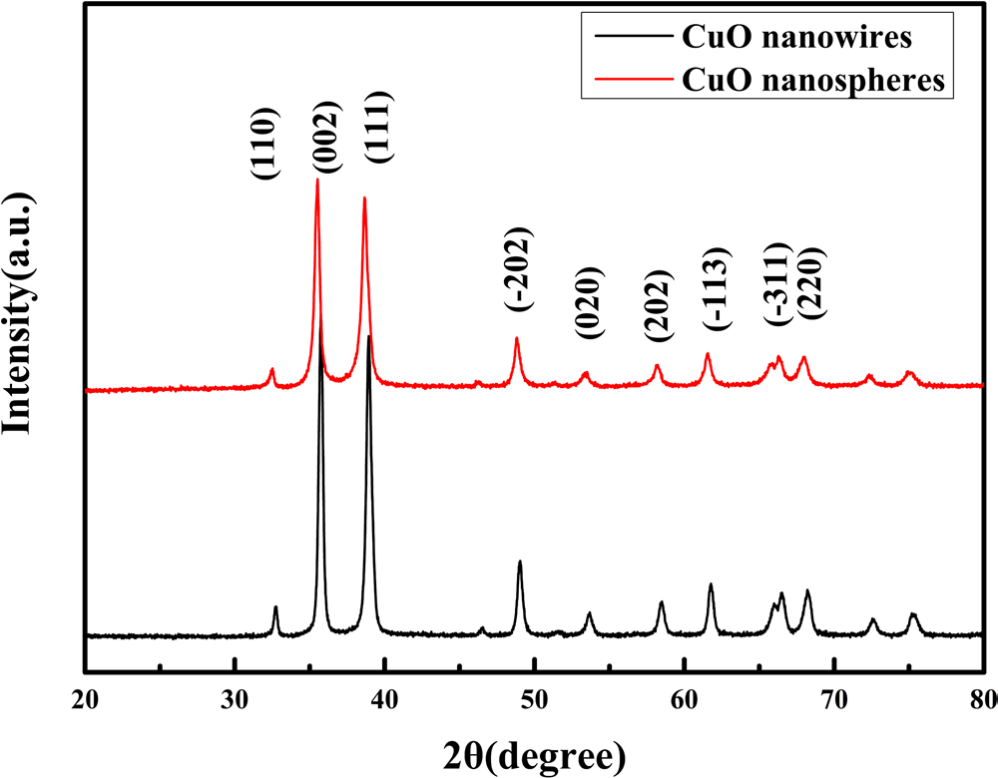
XRD patterns of CuO nanowires and CuO nanospheres.

In order to analyze the inside materials of oxidized copper foil, the oxide on the copper foil was carefully brushed off by a brush for XRD testing. The main characteristic diffraction peaks of the two samples are consistent(Fig. 4), and the corresponding 2θ is also consistent, indicating that the two samples have the same phase. Consistent with the peaks of the copper oxide standard PDF#48-1548, 32.6° CuO 110 peak; 35.7° CuO 002 peak; 38.9° CuO 111 peak; 49.0° CuO -202 peak; 53.6° CuO 020 peak; 61.7°, CuO -113 peak; 66.2°, CuO -311 peak; 68.4 CuO 220 peak; The peaks of CuO were determined to be pure copper oxide, and the diffraction peaks of the samples were sharp, which indicates that the CuO is monoclinic.

**Figure 5:**
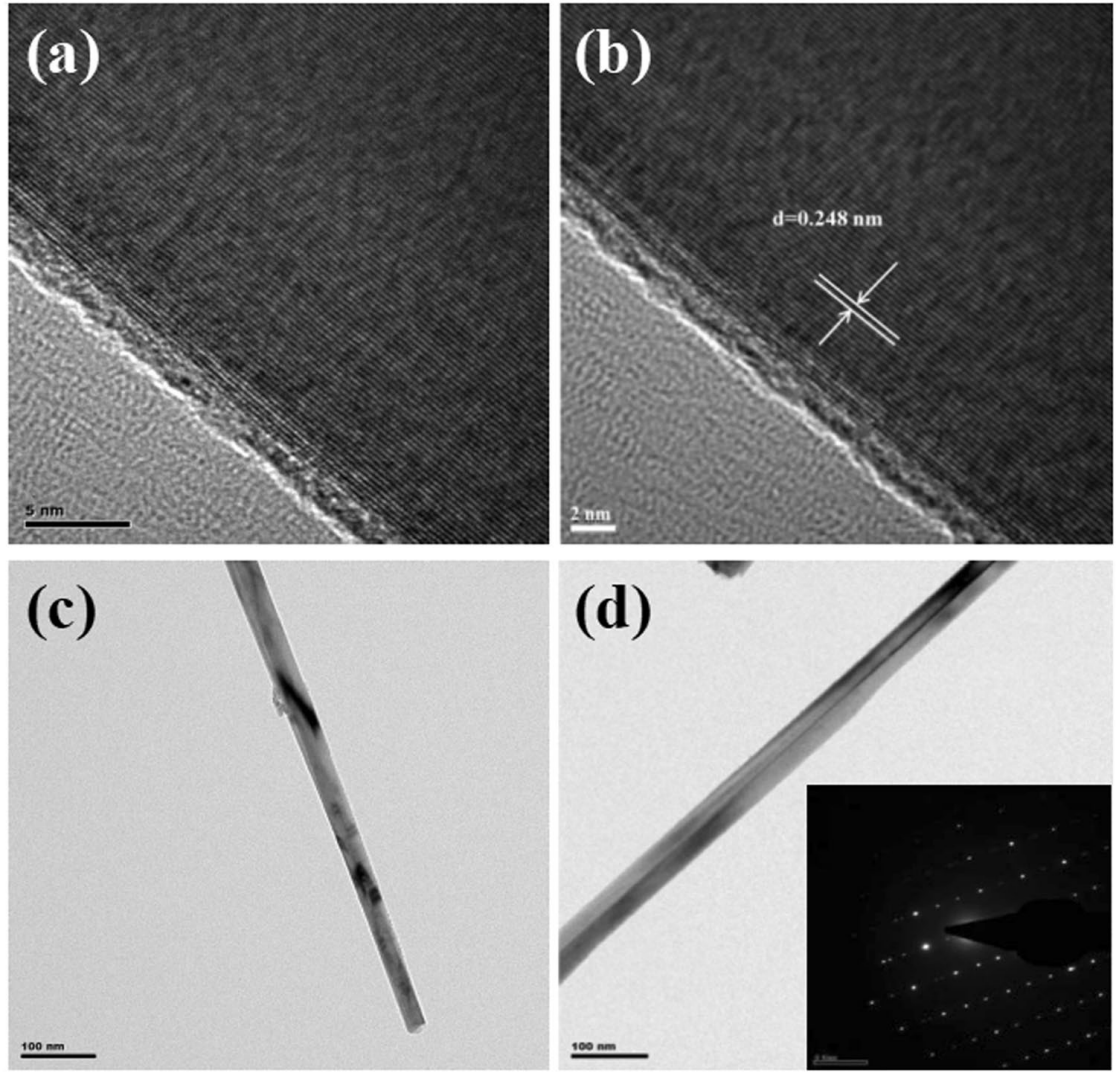
(**a**,**b**) TEM image, selected-area electron diffraction, (**c**,**d**) HRTEM image of one single CuO nanowire.

The structure of the nanowires was studied by transmission electron microscopy (TEM). Fig. 5 displays a single nanowire, which shows multi-face and the edges are clear, CuO nanowires are not empty inside, but solid. It is consistent with the SEM result as shown in Fig. 1. The SAED pattern indicates that the nanowires are CuO with a monoclinic structure. A high-resolution TEM (HRTEM) image clearly shows that the nanowire is single crystalline CuO. The d-space of nanowire calculated from the corresponding SAED pattern is 0.248 nm, which corresponds to its (111) plane. The SAED pattern indicates that the nanowire is monocrystalline with a monoclinic structure.

### Comparison of the enhanced thermal conductivity of nanofluids

**Figure 6:**
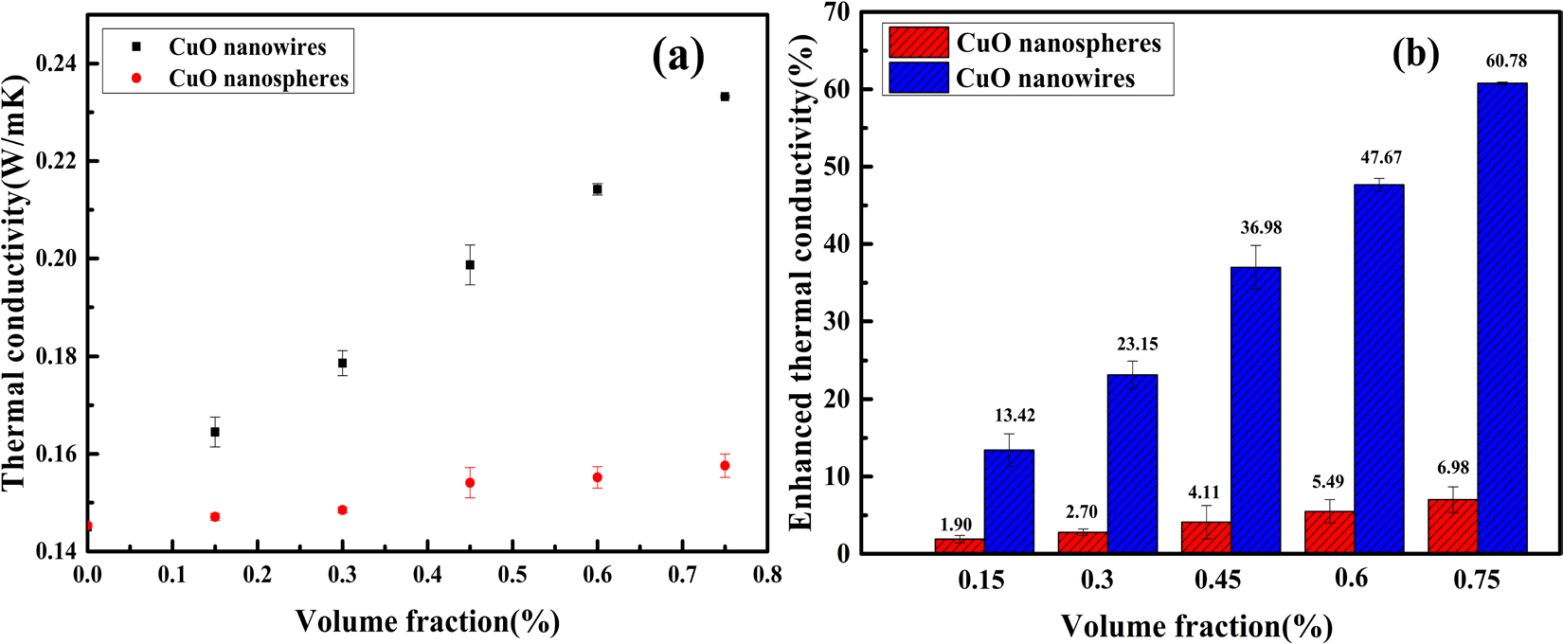
Thermal conductivity (**a**) and enhanced thermal conductivity of nanofluids (**b**) with different CuO particles as a function of the volume fraction.

Thermal conductivity of nanofluilds were measured by a thermal conductivity analyzer at the temperature of 25 °C. The experimental data has shown that CuO nanowires contained nanofluids have higher thermal conductivity. The thermal conductivity of base fluid is only 0.145 W/mK, while the CuO nanowires and nanospheres are well dispersed in dimethicone, the suspensions are stable. Seen from Fig. 6, the volume fraction of CuO particles ranges from 0 to 0.75%. It can be observed that the thermal conductivity of nanofluid increases with the volume fraction of CuO. At the volume fraction of 0.75%, the thermal conductivity of the nanofluids containing CuO nanowires and nanospheres are 0.2332 W/mK and 0.1552 W/mK, respectively. For CuO nanospheres, the thermal conductivity enhancement is 6.98%. While it is intriguing that a maximum increase in thermal conductivity of nanofluid including CuO nanowires reaches up to 60.78%. No matter the nanofluids contain CuO nanowires or nanospheres, there is a nearly linear relationship between the thermal conductivity and volume fraction. The thermal conductivity of the nanofluids is enhanced because the thermal conductivity of the solid particles is much larger than that of the liquid. When the CuO nanowires and CuO nanospheres are added into the base fluids, it will change the structure of the liquid. The energy transfer process inside the mixture will increase, so the integral thermal conductivity will be increased.

By comparison, we can draw a conclusion that the thermal conductivity of CuO nanowires contained nanofluids is much higher than that of CuO nanospheres contained nanofluids at the same conditions in our experiment. Compared to other work, it is an intriguingly high thermal conductivity increment at low loading using CuO nanowires as the thermal additive. Lee *et al*. investigated the property of the suspension of 4.0 vol.% 35 nm CuO particles in ethylene glycol and observed a 20% increase in thermal conductivity^29^. Agarwal *et al*. made a series of experiments about CuO nanofluilds, and they declared that the thermal conductivity of nanofluilds increased by 40%, 27%, 19% using distilled water, ethylene glycol, and engine oil as the base fluidsrespectively^30^. Manimaran *et al*. prepared CuO nanofluids by the single-step wet chemical precipitation method^31^. A maximum increase in thermal conductivity of the CuO nanofluid was found to be 12.4% compared to deionised water. Karami *et al*. had studied the thermo-optical properties of CuO nanofluids for direct absorption of solar radiation, and got about a 13.7% thermal conductivity increase in the base fluid (distilled water:ethylene glycol = 7:3)^32^. Peterson *et al*. experimentally observed the thermal conductivity of CuO nanoparticles in water^33^, and they reported that a 52% increment on thermal conductivity at 6 vol.%. Nemade *et al*. tried to use the ultrasound method to improve the thermal conductivity of nanofluids^34^, and the CuO/H_2_O nanofluids achieved an 18% enhancement in thermal conductivity for the 60 min of probe sonication time. Yu *et al*. had studied the thermal conductivity enhancement in thermal grease containing different CuO structures. Compared with pure silicone base, the thermal conductivity of thermal greases with CuO microspheres increases 8.3% at filler loading of 1 vol.%^19^. In this work, the thermal conductivity enhancement of CuO nanospheres as thermal additive is consistent with the thermal conductivity data measured by other copper oxide work. But the thermal conductivity of CuO nanowires contained nanofluids is much higher than the work ever reported at the same conditions.

The previous research has shown that the thermal conductivity of nanofluids is determined by many factors^5^, including the type of nanoparticles, base fluids and temperature. The effects of particle include: concentration, agglomeration, size shape and surface charge. The effects of base fluids include the thermal conductivity of base fluids and the viscosity. To explain the reasons for the increase of the thermal conductivity in nanofluids, the heat transfer mechanisms in nanofluids have been proposed by many scientists. The effects of the particle-fluid interfacial layering, particle aggregation, and particle Brownian motion have been considered^35–37^. The mechanism of interfacial layering^35^ argues that the liquid molecules that near surface of nanoparticles will form a layered structure, so the liquid molecules near the interface of the contact solid are arranged more orderly than the inside of the liquid, which is similar to the solid phase structure, and it has better heat transfer performance than the base liquid. The solid-like liquid layers act as thermal bridges between the bulk liquid and solid particles, and it will lead the thermal conductivity to increase.

It was first conceptualized by Keblinski *et al*. that thermal conductivity of nanofluidscan be enhanced by clustering/aggregation of nanoparticles^37^. Because of the Van der Waals attractive force, small particles have a tendency to form aggregates in the base fluid. There are clusters of nanoparticles that are small but dispersible and stable in the suspension. If the nanoparticle spacing is small, the liquid film layers that attached to the two particles will contact or even partially overlap, so that the two nanoparticles will contact with each other directly. It leads to thermal short circuit and greatly reducing heat resistance, and then the effective thermal conductivity of the nanofluids will increase. The effects of Brownian motion^36^ was explained like these: when the particle size is large, the Brownian motion is very small, and the Van der Waals force can be negligible, but when the particle size is not small, Brownian motion cannot be ignored and it will increase the collision frequency between the particles and the particles, causing the particles to accumulate and produce micro-convection between the particles and the liquid. Therefore, the thermal conductivity of the nanofluids is determined by the effective thermal diffusion and particle migration of the solid-liquid two phases.

Although so many mechanisms are proposed, there are no general mechanisms to rule the strange behavior of nanofluids including the highly improved effective thermal conductivity. But these mechanisms have a meaningful reference for us to explain the reasons for the increase in thermal conductivity of nanofluids.

As all we know, metal oxideshave important influence on the thermal conductivity of nanofluids. We believe that there is a very significant relationship between the structure of copper oxide and thermal conductivity of nanofluids. As a matter of fact, the original intrinsic properties of metal nanostructures are determined by its size, structure, and mutual interaction between nanoparticles^38^. The CuO nanospheres are zero-dimensional (0-D) nanomaterials and CuO nanowires are one-dimensional (1-D) nanomaterials. Some scientists have proved the advantage of one dimensional material in heat transfer. Compared with nanoparticles, 1-D CuO nanowires have smaller dimension structure, and high aspect ratio, which could efficiently transport thermal carriers along one controllable direction^39–41^.

Besides, surface phonon is also one of the causes of thermal conductivity. The heat conduction of the solid material is mainly realized by the lattice vibration (phonon). When the temperature is not too high, the heat conduction is mainly phonon conduction. The relationship can be represented as follow:

1 $$\lambda =\frac{1}{3}Cvl$$

where λ is the thermal conducticity, ν is the phonon frequency, C is the volumetric heat capacity per unit phonon frequency, and l is the phononmean-free-paths. CuO nanowires also have long phonon-mean-freepaths compared with nanoparticles, which will contributions to the higher thermal conductivity. When copper oxide is added and stably dispersed in dimethicone, the thermal conductivity of the two phases differs greatly, then the heat is mainly transferred by the phase having a high thermal conductivity. It also explains that the suspensions with CuO nanorods and nanowires always displayed higher thermal conductivity than that with CuO nanospheres. Therefore, it is concluded that the shape factor has a vital influence on thermal conductivity of nanofluids^42^.

### Theoretical models of thermal conductivity

Many equations have been proposed for the transport properties, such as electrical and thermal conductivity of two-phase systems. The effective theoretical model can predict and guide the experimental results. The thermal conductivity of different morphology CuO nanofluids were further calculated by comparing our experimental results with existing theoretical models. The experimental data were compared with Maxwell^43^, Bruggeman^44^ and Hamilton-crosser^45^ model prediction, the influence mechanism were discussed as well.

Maxwell model is famous for predicting the thermal conductivity of dilute suspension with large and spherical particles. It could be represented as follow:

2 $${k}_{C,Maxwell}={k}_{b}[\frac{{k}_{p}+2{k}_{b}+2V({k}_{p}-{k}_{b})}{{k}_{p}+2{k}_{b}-V({k}_{p}-{k}_{b})}]$$

Bruggeman proposed a model to analyze the interactions among randomly distributed particles. For a binary mixture of homogeneous spherical inclusions, it can be represented as follow:

3 $${k}_{C}=\frac{{k}_{b}}{4}[{(3V-1)}^{2}(\frac{{k}_{p}}{{k}_{b}})+(2-3V)+\sqrt{{\rm{\Delta }}}]$$

4 $${\rm{\Delta }}=[{(3V-1)}^{2}{(\frac{{k}_{p}}{{k}_{b}})}^{2}+{(2-3V)}^{2}+2(2+9V-9{V}^{2})\frac{{k}_{p}}{{k}_{b}}]$$

Maxwell model and Bruggeman model ignore the effects of particles size, morphology and other factors. As a matter of fact, particle size and shape of fillers usually affect the coefficient of theoretical models. On the basis of the Maxwell model, Hamilton and Crosser take into account the shape of the particles. The Hamilton-crosser model, it could be represented as follow:

5 $${k}_{C}={k}_{b}[\frac{{k}_{p}+({\rm{n}}-1){k}_{b}+(n-1)V({k}_{p}-{k}_{b})}{{k}_{p}+(n-1){k}_{b}-V({k}_{p}-{k}_{b})}]$$

In these theoretical models, k_c_, k_b_ and k_p_ represent the thermal conductivity of the system, base fluid and filler. V is the volume fraction of the fillers. n = 3/Ф and Ф is the sphericity of the filler particle, n is empirical shape factor. For the spherical particle, the sphericity (Ф) is 1, n = 3, so Hamilton-crosser model is equal to Maxwell model. For the dimethicone composites containing CuO nanowires, the k_b_ and k_p_ are set to 0.145 W/mK and 69 W/mK^46^. Considering the discontinuous phase particle shape and dimension of principal axis direction, Yamada *et al*. modified this theory model based on the unit-cell model^47^. That formula is as follow:

6 $${k}_{C}={k}_{b}[\frac{{k}_{p}+K{k}_{b}+KV({k}_{p}-{k}_{b})}{{k}_{p}+K{k}_{b}-V({k}_{p}-{k}_{b})}]$$

where K is the shape factor, K = 2Ф^0.2^(l_p_/l_d_), l_p_ and l_d_ are the length and diameter of the particle, respectively. K is equivalent to (n − 1). It’s easy to calculate that l_p_/l_d_ = 71.32, n = 75.78.

**Figure 7:**
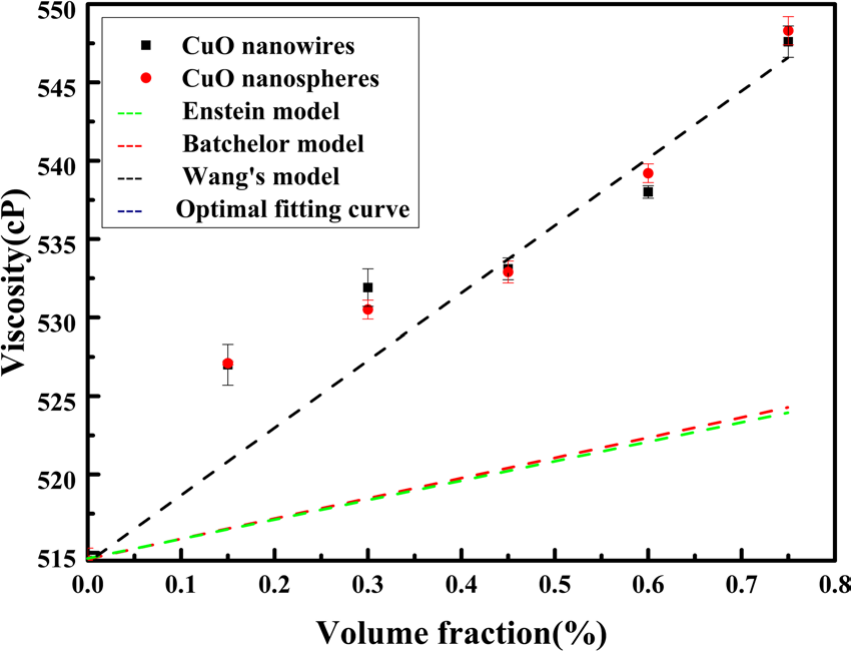
Analysis of prediction model and experimental data.

According to prediction model, for CuO nanospheres, the sphericity (Ф) is 1 and the values of Maxwell model show basically agreement with the experimental results of CuO nanospheres. Additionally, the thermal conductivity of Bruggeman model’s theoretical prediction value is much higher than the experimentally determined values. It cannot predict the experimentally determined values accurately. Maxwell model shows a better prediction than Bruggeman model, so Maxwell model is found to be suitable and can give better predictions for the thermal conductivity of CuO nanosphere contained nanofluids. For CuO nanowires, the shape of the particles must be taken into account. The sphericity (Ф) is 0.04, and it is observed from Fig. 7 that the obtained experimental thermal conductivity values are close to the theoretical predictions by the Hamilton–Crosser.

### Viscosity of nanofluids

In the last two decades, many studies have been performed on effective viscosity. The other physical properties of fluids may change when nanoparticles are added into the base fluid. In the applications of heat transfer for nanofluids, viscosity is as important as thermal conductivity^48^ and it will influence the flow and heat transfer characteristics. Viscosity describes the internal resistance of fluid flow and is used to evaluate the pumping power, which affects the pressure drop and enhances the pumping power^49^ when nanofluids are circulated in a closed loop for transfer of heat in heat exchangers^50^.

**Figure 8:**
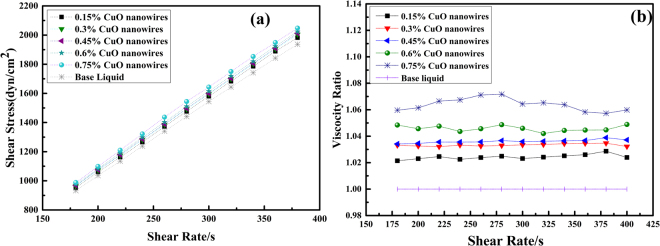
(**a**) Shear stress and shear strain relations for various volume concentrations of CuO nanofluid; (**b**) Viscosity ratio vs shear rate for different volume concentration of CuO nanofluids.

Fig. 8(a) shows the trend of shear stress as a function of the shear strain for CuO nanowires contained nanofluids. For different volume concentration of CuO nanofluids, there is a linear relationship between the shear stress and shear rate, demonstrating that the CuO nanowires contained nanofluids behave as Newtonian fluid at the tested conditions. For easy calculation, viscosity ratio is defined as the ratio of the viscosity of the nanofluid to that of the base fluid. The viscosity ratio of the nanofluids as a function of shear strain rate is shown in Fig. 8(b). The shear stress does not vary with shear rate, indicating Newtonian behavior too, and it is observed that the viscosity ratio increases with volume fraction of CuO nanowires.

**Figure 9:**
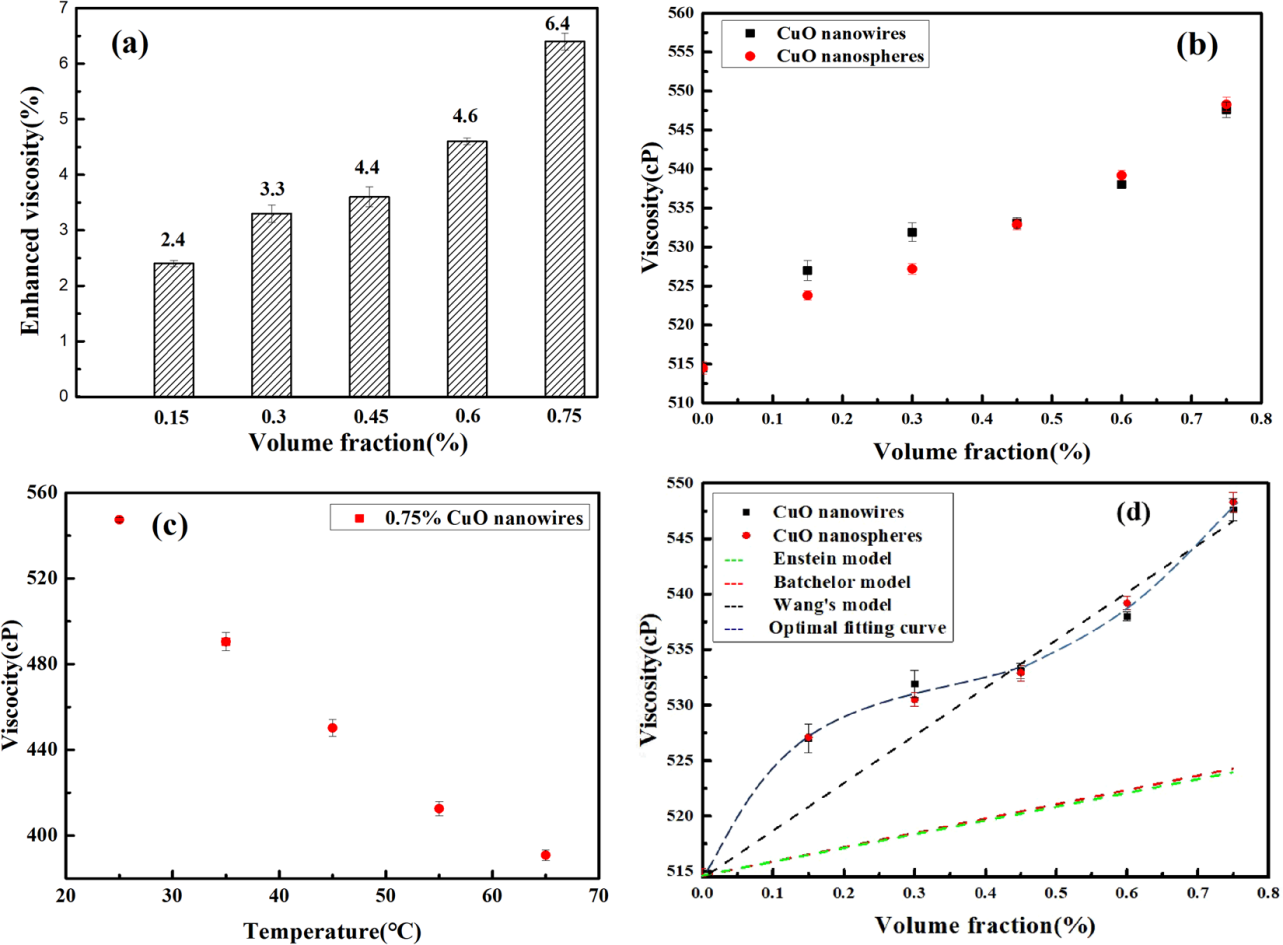
(**a**) The average viscosity ratio and enhanced for various volume concentrations of CuO nanofluids. (**b**) Viscosity of nanofLluids with different CuO particles as a functionof volume fraction, (**c**) Viscosity of nanofluids containing 0.75% CuO nanowires at various temperature, (**d**) Compared with the predictions from different classical and empirical viscosity models.

The results of average viscosity increase with the volume concentrations of CuO nanofluid are shown in Fig. 9(a), and our measured viscosity of CuO based nanofluids are found to maximal increase by nearly 6.41% at the volume fraction of 0.75%. The increment of viscosity by adding more nanoparticles is caused by the increase of fraction and flowing resistance of the nanofluids^51^. When the nanofluids flow, in order to overcome the internal friction resistance, it needs to consume a certain amount of energy. The more particles in the nanofluids, the more energy consumption, so that the greater the volume fraction of nanoparticles, the higher the viscosity of the nanofluids.

Murshed *et al*. experimentally studied the viscosity of the nanofluids^52^, and they concluded that the measured viscosity of Al_2_O_3_/water based nanofluids were found to increase by nearly 82% for the maximum volumetric loading of nanoparticles 5%. Chiam *et al*. investigated the thermal conductivity and viscosity of Al_2_O_3_ nanofluids for different based ratio of water and ethylene glycol mixture^53^, and showed that the average dynamic viscosity enhanced up to 50% at concentrations from 0.2 to 1.0% for a mixture of water:EG at 60:40. The effect of temperature and nanoparticles volume fraction on the viscosity of copper oxide-ethylene glycol nanofluids were experimentally studied by Esfe *et al*., who found that the maximum increase relative viscosity is 82.46% that occurs in a volume fraction of 1.5% and temperature of 50 °C^51,54^.

Compared with above work, the increase of viscosity for CuO nanowires contained nanofluids is lower than that of the other solution at the same concentration. It means that the nanofluids system has a good liquidity, which may be the result of the combined action of the base fluids and nanoparticles^55^. The low viscosity is of great significance for the flow of fluid and the process of heat transfer and mass transfer^56^. The larger the viscosity of the fluid in the same flow condition, the greater the resistance of the fluid. Therefore, the nanofluids are not suitable for larger viscosity during the enhanced heat transfer process. Due to the low viscosity, the motion of particles in dimethicone will be more intense and the molecular force will be reduced, and fluid becomes easier to move. So the CuO nanowires contained nanofluids has its great advantages because of its low viscosity.

Up to date, a few studies have investigated and proved the effect of nanoparticles’ shape on the viscosity of nanofluids, but there is not a consistent conclusion. For ZnO/water suspensions, Ferrouillat *et al*. found that the viscosity of nanofluids with rod-shaped nanoparticles is slightly less than that with polygonal particles^57^. Timofeeva *et al*. studied the particle shape effect on thermophysical properties of alumina nanofluids, they thought that the viscosities of nanofluids presented such a relationship: blades < bricks < cylinders < platelets at the same particle concentration^58^. In our study, Fig. 9(b) shows the increasing trend of viscosity with the rise in volume fraction, we can find that the shape of the particles has no obvious effect on the viscosity of the nanofluids at the same condition. This may be due to the low concentration of nanoparticles.

As it shows in Fig. 9(c), we studied the effect of temperature on viscosity, the experimental findings exhibit that the viscosity of 0.75% CuO nanofluids reduces with the increase of temperature. At the temperature of 65 °C, the viscosity of the CuO nanowires contained nanofluids decreases by 40%. Many scientists have interpreted the reasons. With the increase of temperature, the Brownian motion of CuO nanoparticles will increase in base fluid. The increase in the random velocity of the nanoparticles results in a decrease in the intermolecular forces between the base fluid and the nanoparticle surface so that the viscosity of the nanofluid will be lower at higher temperatures.

### Theoretical models of viscosity

Einsteinhas proposed a viscosity correlation in terms of nanoparticle concentration in the base fluid. This model was stated to be valid for solid when the nanoparticle volume percentageis lower than 2%. Combined with the Einstein model, so far, many scientists put forward different prediction models (Table 1). However, there is no accurate calculation of various nanofluid viscosity models. This is caused by various factors. The existing models for the calculation of viscosity are evolved from the Einstein viscosity model. From Fig. 9(d), we can find that the classical models are unable to accurately predict the viscosity of nanofluids. Wang’s model is closer to our experimental data.

**Table 1:** Typical nanofluid viscosity prediction models.

Researcher	Year	Model	R^2^	Remarks
Einstein^63^	1906	*μ*_*s*_ = *μ*_bf_(1 + 2.5*φ*)	0.501	For rigid solid spheres, the volume fraction <2%
Batchelor^64^	1977	*μ*_*s*_ = *μ*_bf_(1 + 2.5*φ* + 6.2*φ*^2^)	0.5312	Interactions between particles was considered, a very low concentration
Wang *et al*.^65^	1999	*μ*_*s*_ = *μ*_bf_(1 + 7.3*φ* + 123*φ*^2^)	0.8351	Empirical generic model
This study	2017	*μ*_*s*_ = *μ*_bf_(1 + 117*φ* *− *2*69*.*8φ*^*2*^* + 229*.*6φ*^3^)	0.9972	Empirical model

We tried to use the prediction models to verify the available experimental data. Combined with the experimental data, the existing viscosity models are amended to obtain a new viscosity calculation formula to satisfy the following equation:

7 $${\mu }_{s}={\mu }_{bf}(1+a\phi +b{\phi }^{2}+c{\phi }^{3})$$

In this theoretical mode, µ_s_ = suspension viscosity, µ_bf_ = viscosity of base fluid, *a*, *b*, *c* is constant, and *φ* is volume concentration of particles in base fluid. Here, *a* = 117.4, *b* = −269.8, *c* = 229.6, *R*^2^ = 0.99718. Our empirical model shows basically agreement with the experimental results at the same volume fraction.

The experimental measurements and theoretical predictions on the viscosity of nanofluids are still in the analysis stage, and the experimental results of viscosity in the different literature are not consistent^59^. The empirical models are also not suitable for the predictions of viscosity of other types of nanofluids. So, it is vital to put forward a universal theoretical model that can take into account all potential factors for the predictions of viscosity of any nanofluids.

## Conclusion

In this work, CuO nanowires and nanospheres were prepared through thermal oxidation method and chemical method, respectively. CuO nanofluids were further obtained by dispersing CuO nanowires and nanospheres into dimethicone under sonication. The thermal conductivity and viscosity of dimethicone based CuO nanofluids have been experimentally and theoretically investigated. We have obtained a high thermal conductivity increment at low loading using CuO nanowires as the thermal additive in nanofluids. Experimental data have shown that the as prepared CuO nanowires contained nanofluids have intriguingly higher thermal conductivity than the previously reported CuO nanofluids. We have found that the thermal conductivity of nanofluid increases nearly linearly with the volume fraction of particles and a maximum increase reached 60.78% at very low loading of 0.75% in volume fraction. In addition, the results show that the nanofluids have Newtonian behaviors under the condition of this work, and they have low enhanced viscosity. The measured viscosity of CuO based nanofluid has only a 6.41% maximal increase at the volume fraction of 0.75%. The mechanisms of thermal conductivity and viscosity were also discussed as well. The effect of copper oxide nanowires on the thermal conductivity of nanofluids are proved and verified.

## Methods

### Materials

All the reagents were purchased from Sinopharm Chemical Reagent Co., Ltd., Shanghai, China, and they were all analytical grade and used without further purification.

### Synthesis of CuO nanowires

**Figure 10:**
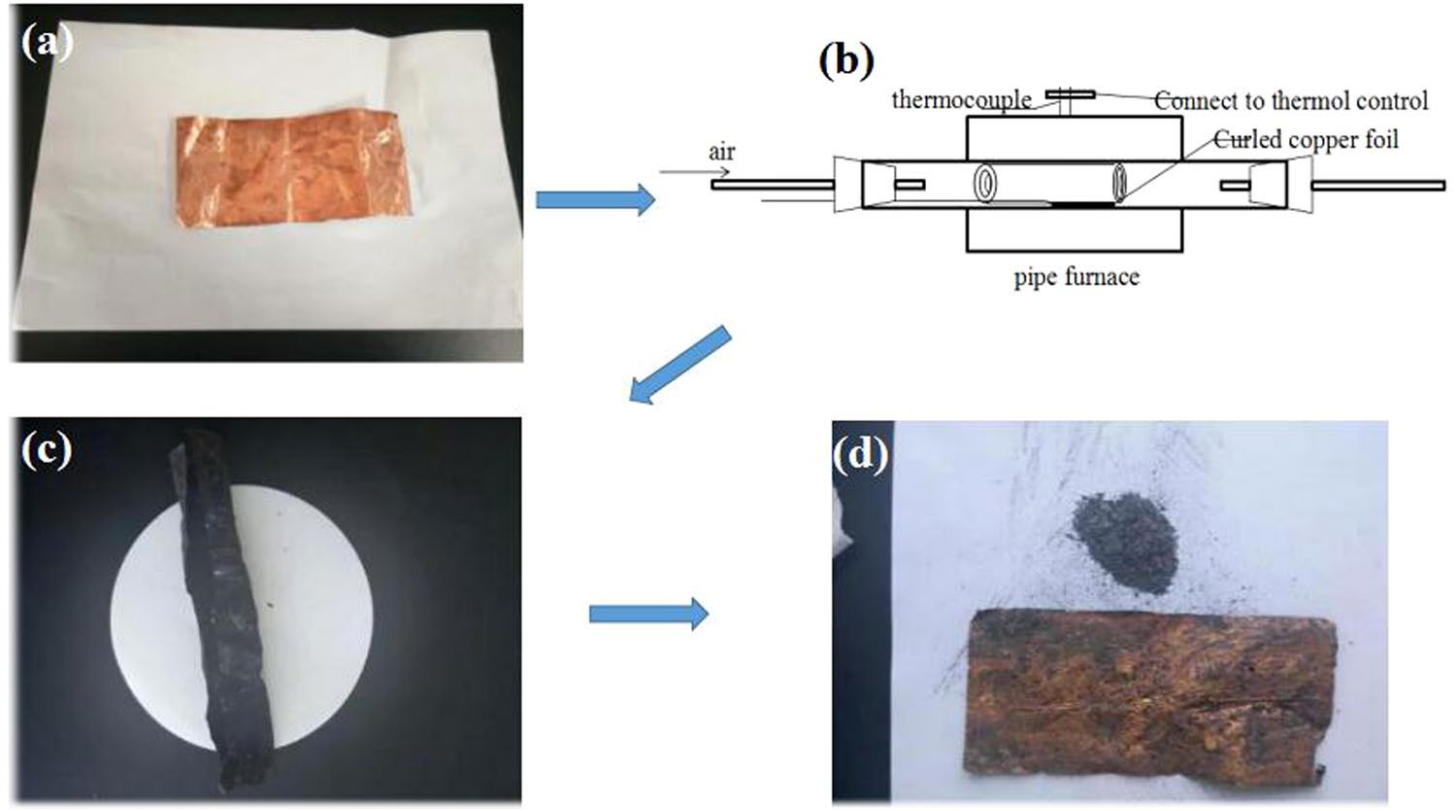
Preparation process of CuO nanowires through thermal oxidation method, (**a**) The pure copper foil, (**b**) Copper foil calcination process, (**c**) The calcined copper foil, (**d**) The black copper oxide nanowires peeled off from the surface of copper foil.

In the present work, we used thermal oxidation method to prepare CuO nanowires^60^ with some modifications. The preparation processesare shown in Fig. 10. CuO nanowires grow on the Cu foil perpendicularly. The root of CuO nanowires is thick and the tip is thin. The oxide layer contains different oxides, with CuO nanowire, layer of CuO, Cu_2_O layer from top to bottom. The CuO nanowires were prepared by three steps. Firstly, let the copper foil soak for 4 hours in dilute hydrochloric acid solution (1M), and then repeated washing with deionized water until the washing solution is neutral. This step will remove the surface oxides and impurities. The second step is the thermal oxidation process. The Cu foil will be heated under air atmosphere to 400 °C with a heating rate of 3 °C min^−1^. Lastly, the black oxide will be peeled off from the surface of copper foil. We use a brush to carefully brush off the oxide, and the black oxide is the copper oxide nanowire powder.

### Preparation of CuO nanospheres

CuO nanospheres were synthesized according to the method reported by Jia *et al*.^61^. Some modification has been done in this work. Firstly, a solution containing copper acetate (0.015 M) and urea (0.015 M) was placed in Teflon-lined stainless steel autoclave and maintained at 120 °C for 5 h. Then, it was cooled to room temperature and a black precipitate was obtained. Next, the solid product was recovered by adding deionized water and absolute ethyl alcohol centrifuged at 10000 rpm for 5 min, repeated for three times respectively. Lastly, it was dried in an oven at 80 °C for 12 h.

### Preparation of CuO nanofluids

We prepared the CuO nanofluids by a so called “two-step method”. Firstly, the CuO nanowires and spheres were directly added into the base liquid. Then, followed by an ultrasonic dispersion to interrupt the hard agglomeration between the nanoparticles, a stable and well dispersed nanofluid can be obtained.

### Characterization

A scanning electron microscopy (SEM, Hitachi S4800) was used to examine the dimension and shape of CuO nanowires and CuO nanospheres. Phase composition and crystallinity of CuO nanowires and CuO nanospheres were recorded using a X-ray diffractometer (XRD, D8-Advance, Germany) with a back monochromator operating at 40 kV and a copper cathode as the X-ray source (λ = 0.154 nm). XRD patterns were recorded from 10 to 80° (2θ) with a scanning step of 0.01°. A thermal conductivity analyzer from Tci^TM^/C-Therm was used to measure the thermal conductivity of nanofluids. A spiral-type heating source is located at the center of the sensor, and heat is generated at the center. The heat that has been generated enters the material through the sensor during which a voltage decrease occurs rapidly at the heating source, and the thermal conductivity is calculated through the voltage decrease data. The testing capabilities of the system is 0 to 100 W/mK across a wide range of temperature (−50 to 200 °C). The accuracy of these measurements was estimated to be within ±5%. This instrument is a state of the art thermal property characterization instrument based on the modified transient plane source (TPS) technique. The samples will be tested 5 times to obtain the average value. The temperature of test system was controlled at 25 °C by constant temperature box (Shanghai Boxun Industry & Commerce Co., Ltd.). The viscosities of CuO nanofluids were measured by DV2T viscometer (HV, Brookfield Engineering Labs., Inc., USA). A standardized spring can drive a rotor. Fluid viscous resistance on rotor was measured by the spring deformation degree. Circulation constant temperature water bath was used to keep the samples at 25 °C. Shear viscosities of nanofluid were measured range from 180 to 400 s^−1^.

## References

[1] Chol S (1995). Enhancing thermal conductivity of fluids with nanoparticles[J]. ASME-Publications-Fed.

[2] Girishkumar GS, Nataraj SCN (2016). Experimental investigation of copper oxide (CuO) nanofluid on cylindrical heat pipe thermal performance[J]. International Research Journal of Engineering and Technology.

[3] Trisaksri V, Wongwises S (2007). Critical review of heat transfer characteristics of nanofluids [J]. Renewable & Sustainable Energy Reviews.

[4] Khurana D, Choudhary R, Subudhi S (2017). A critical review of forced convection heat transfer and pressure drop of Al_2_O_3_, TiO_2_, and CuO nanofluids [J]. Heat & Mass Transfer.

[5] Lee JH (2011). A Review of Thermal Conductivity Data, Mechanisms and Models for Nanofluids[J]. International journal of micro-nano scale transport.

[6] Li H (2015). Experimental investigation of thermal conductivity and viscosity of ethylene glycol based ZnO nanofluids [J]. Applied Thermal Engineering.

[7] José PGM (2011). Thermal conductivity and viscosity measurements of ethylene glycol-based Al_2_O_3_nanofluids [J]. Nanoscale Research Letters.

[8] Vasheghani M (2013). [J]. Nanomechanics Science & Technology An International Journal.

[9] Yu W (2009). Investigation of thermal conductivity and viscosity of ethylene glycol based ZnO nanofluid [J]. Thermochimica Acta.

[10] Ahmed J, Mao Y (2016). Synthesis, characterization and electrocatalytic properties of delafossite CuGaO_2_[J]. Journal of Solid State Chemistry.

[11] Ahmed J (2016). Sol – gel synthesis, structural characterization and bifunctional catalytic activity of nanocrystalline delafossite CuGaO_2_, particles[J]. Journal of Alloys & Compounds.

[12] Park E, Park HW, Lee J (2015). Synthesis of hierarchical copper oxide composites prepared via electrical explosion of the wire in liquids method [J]. Colloids & Surfaces A Physicochemical & Engineering Aspects.

[13] İbrahim Y, Erdoğan ÖG (2010). Optical and structural properties of CuO nanofilm: Its diode application [J]. Journal of Alloys & Compounds.

[14] Qiu G (2012). Facile Microwave-Assisted Hydrothermal Synthesis of CuO Nanomaterials and Their Catalytic and Electrochemical Properties [J]. Journal of Physical Chemistry C.

[15] Xiang JY (2010). Self-assembled synthesis of hierarchical nanostructured CuO with various morphologies and their application as anodes for lithium ion batteries [J]. Journal of Power Sources.

[16] Guan X (2011). Hierarchical CuO hollow microspheres: Controlled synthesis for enhanced lithium storage performance [J]. Journal of Alloys &Compounds.

[17] Sivakumar A, Alagumurthi N, Senthilvelan T (2014). Experimental investigation in thermal conductivity of CuO and ethylene glycol nanofluid in serpentine shaped microchannel[J]. International Journal of Engineering Science and Technology.

[18] Takahashi K (2009). Thermal conduction of one-dimensional materials [J]. Thermophysical Properties.

[19] Zhao JC (2015). Thermal conductivity enhancement in thermal grease containing different CuO structures [J]. Nanoscale Research Letters.

[20] Mema R (2011). Effect of surface stresses on CuO nanowire growth in the thermal oxidation of copper [J]. Chemical Physics Letters.

[21] Mimura K (2006). Brief review of oxidation kinetics of copper at 350 °C to 1050 °C [J]. Metallurgical & Materials Transactions A.

[22] Zhong ML (2010). Synthesis, growth mechanism and gas-sensing properties of large-scale CuO nanowires [J]. Acta Materialia.

[23] Chen JT (2008). CuO nanowires synthesized by thermal oxidation route [J]. Journal of Alloys & Compounds.

[24] Zhong W (1998). Growth units and forming mechanism of KDP crystals [J]. Science in China.

[25] Berry LG, Mason BH (1983). Mineralogy:concepts, descriptions, determinations[M]. W. H. Freeman and company.

[26] Luo, Y., Xu, N.S. Thermodynamic mechanism responsible for growth of CuO nanowires by thermal oxidation [C]// Vacuum Nanoelectronics Conference. IEEE, 173–174 (2010).

[27] Xu CH, Woo CH, Shi SQ (2004). Formation of CuO nanowires on Cu foil [J]. Chemical Physics Letters.

[28] Adilov SR (2017). Studying the composition and structure of films obtained by thermal oxidation of copper [J]. Glass Physics & Chemistry.

[29] Lee SP (1999). Measuring Thermal Conductivity of Fluids Containing Oxide Nanoparticles [J]. Journal of Heat Transfer.

[30] Agarwal R (2016). Synthesis, characterization, thermal conductivity and sensitivity of CuO nanofluids [J]. Applied Thermal Engineering.

[31] Manimaran R (2014). Preparation and characterization of copper oxide nanofluid for heat transfer applications [J]. Applied Nanoscience.

[32] Karami M (2016). Thermo-optical properties of copper oxide nanofluids for direct absorption of solar radiation [J]. Solar Energy Materials & Solar Cells.

[33] Li CH, Peterson GP (2006). Experimental investigation of temperature and volume fraction variations on the effective thermal conductivity of nanoparticle suspensions (nanofluids) [J]. Appl Phys.

[34] Nemade K, Waghuley S (2016). A novel approach for enhancement of thermal conductivity of CuO/H_2_O based nanofluids [J]. Applied Thermal Engineering.

[35] Yu W (2010). Mechanisms and models of effective thermal conductivities of nanofluids[J]. Journal of Nanoscience & Nanotechnology.

[36] Xie H (2002). Dependence of the thermal conductivity of nanoparticle-fluid mixture on the base fluid [J]. Journal of Materials Science Letters.

[37] Keblinski P (2002). Mechanisms of heat flow in suspensions of nano-sized particles (nanofluids)[J]. International Journal of Heat & Mass Transfer.

[38] Hua YC, Cao BY (2016). The effective thermal conductivity of ballistic–diffusive heat conduction in nanostructures with internal heat source[J]. International Journal of Heat & Mass Transfer.

[39] Hu J, Odom TW, Lieber CM (1999). Chemistry and physics in one dimension: synthesis and properties of nanowires and nanotubes[J]. Accounts of chemical research.

[40] Wang ZL, Dai Z, Sun S (2000). Polyhedral Shapes of Cobalt Nanocrystals and Their Effect on Ordered Nanocrystal Assembly[J]. Advanced Materials.

[41] Xia Y (2003). One-dimensional nanostructures:synthesis, characterization, and applications [J]. Advanced Materials.

[42] Zhu D (2016). Thermal Conductivity of Composite Materials Containing Copper Nanowires[J]. Journal of Nanomaterials.

[43] Maxwell JC (1904). A Treatise on Electricity and Magnetism 2 [J]. Nature.

[44] Bruggeman DAG (1935). Calculation of various physical constants of heterogeneous substances, I. dielectric constants and conductivity of the substances from mischkorper isotropic [J]. Annals of Physics Leipzig.

[45] Hamilton RL, Crosser OK (1962). Thermal conductivity of heterogeneous twocomponent systems [J]. I&EC Fundamentals.

[46] Xue Q, Xu WM (2005). A model of thermal conductivity of nanofluids with interfacial shells [J]. Materials Chemistry & Physics.

[47] Yamada E, Ota T (1980). Effective thermal conductivity of dispersed materials [J]. Wärme - und Stoffübertragung.

[48] Nguyen CT (2007). Temperature and particle-size dependent viscosity data for water-based nanofluids – Hysteresis phenomenon [J]. International Journal of Heat & Fluid Flow.

[49] Aladag B (2012). Experimental investigations of nanofluids at low temperatures [J]. Applied Energy.

[50] Naik MT (2010). Experimental investigation into rheological property of copper oxide nanoparticles suspended in propylene glycol- water based fluids [J]. Journal of Engineering & Applied Sciences.

[51] Esfe MH (2015). Mixed-convection flow and heat transfer in an inclined cavity equipped to a hot obstacle using nanofluids considering temperature-dependent properties[J]. International Journal of Heat and Mass Transfer.

[52] Murshed S, Leong KC, Yang C (2008). Investigations of thermal conductivity and viscosity of nanofluids [J]. International Journal of Thermal Sciences.

[53] Chiam HW (2017). Thermal conductivity and viscosity of Al_2_O_3_ nanofluids for different based ratio of water and ethylene glycol mixture[J]. Experimental Thermal and Fluid Science.

[54] Esfe MH (2017). The Investigation of Effects of Temperature and Nanoparticles Volume Fraction on the Viscosity of Copper Oxide-ethylene Glycol Nanofluids [J]. Periodica Polytechnica. Chemical Engineering.

[55] Meyer JP (2016). The Viscosity of Nanofluids: A Review of the Theoretical,Empirical, and Numerical Models[J]. Heat Transfer Engineering.

[56] Murshed SMS, Estellé PA (2017). state of the art review on viscosity of nanofluids [J]. Renewable & Sustainable Energy Reviews.

[57] Ferrouillat S (2013). Influence of nanoparticle shape factor on convective heat transfer and energetic performance of water-based SiO_2_, and ZnO nanofluids [J]. Applied Thermal Engineering.

[58] Timofeeva EV, Routbort JL, Singh D (2009). Particle shape effects on thermophysical properties of alumina nanofluids [J]. Journal of Applied Physics.

[59] Meybodi MK (2016). A novel correlation approach for viscosity prediction of water based nanofluids of Al_2_O_3_, TiO_2_, SiO_2_, and CuO [J]. Journal of the Taiwan Institute of Chemical Engineers.

[60] Li A (2014). Copper oxide nanowire arrays synthesized by *in-situ* thermal oxidation as an anode material for lithium-ion batteries [J]. Electrochimica Acta.

[61] Jia W (2009). Spherical CuO synthesized by a simple hydrothermal reaction: Concentration-dependent size and its electrocatalytic application [J]. Materials Research Bulletin.

[62] Xie Ll (2016). Effect of Heat Treatment on the Growth of CuO Nanowires [J]. Journal of Electron Microscopy.

[63] Einstein A (1906). Eine neue bestimmung der moleküldimensionen [J]. Annalen der Physik.

[64] Batchelor GK (1977). The effect of Brownian motion on the bulk stress in a suspension of spherical particles [J]. Journal of fluid mechanics.

[65] Wang X, Xu X, Choi SUS (2012). Thermal Conductivity of Nanoparticle - Fluid Mixture[J]. Journal of Thermophysics & Heat Transfer.

